# Basic Study on Mercury Methylation Under Oxic Conditions: Incubation Experiments Using Seawater from Minamata Bay, Japan

**DOI:** 10.3390/toxics14080717

**Published:** 2026-08-13

**Authors:** Akito Matsuyama, Michiaki Kindaichi, Yoko Taniguchi

**Affiliations:** Department of International Affairs and Research, National Institute for Minamata Disease (NIMD), 4058-18 Hama, Minamata 867-0008, Kumamoto, Japan

**Keywords:** incubation experiment, dissolved methylmercury, dissolved organic carbon, seawater temperature, salinity, mercury methylation, oxic conditions

## Abstract

**Background:** This study investigated the generation of dissolved methylmercury (diss-MeHg) under oxic conditions in the surface waters of Minamata Bay. Although mercury methylation has been extensively investigated under reducing conditions, its occurrence under oxic conditions remains poorly understood. **Methods:** Incubation conditions capable of artificially inducing mercury methylation were established for the first time. Incubation parameters were optimized using surface seawater samples. **Results:** Diss-MeHg production was positively correlated with dissolved organic carbon (DOC) concentrations (1–5 mg/L). The highest methylation was observed at 22 °C and 32.5‰ salinity, though no statistically significant differences were observed within the ranges of 18–22 °C and 31.5–34.5‰. Under the optimized conditions (22 °C, 32.5‰, 5 mg/L DOC), seawater samples were sequentially filtered through membranes of various pore sizes. Diss-MeHg was detected in fractions filtered through 0.8- and 3-µm membrane filters but was absent from the 0.45- and 0.2-µm fractions. Additionally, autoclaving the 0.8 µm filtrate prevented diss-MeHg production. **Conclusions:** These findings suggest the presence of a distinct biological mercury methylation process operating under oxic conditions in surface seawater.

## 1. Introduction

Minamata Bay, Kumamoto Prefecture, Japan, was heavily polluted with mercury discharged by the Chisso Corporation, resulting in the severe degradation of the marine environment. However, the marine environment has improved significantly [1] since the completion of a large-scale dredging project conducted by Kumamoto Prefecture over approximately 14 years (1977–1990).

We have been conducting mercury monitoring in Minamata Bay since 2005 and have reported several findings regarding its environmental conditions. Compared to previously reported dissolved mercury (diss-Hg) concentrations [2], the annual average concentrations measured in Minamata Bay in fiscal year 2024 were 0.42 ± 0.22 ng/L for dissolved total mercury (diss-THg) and 0.04 ± 0.02 ng/L for diss-MeHg [3], representing a decrease to less than one-twentieth of the levels previously reported. However, despite the significant decrease in diss-Hg concentrations in Minamata Bay seawater, studies have reported that the edible muscle of fish from the bay still contains relatively high mercury concentrations [4]. A comparison of the same fish species from other coastal regions of Japan revealed that Hg concentrations were several times higher in Minamata Bay [5]. Furthermore, a study examining individual fish specimens, rather than mean values derived from multiple fish specimens in accordance with governmental methods, reported fish with concentrations exceeding 1 mg/kg [6]. To clarify the causes of these findings, Yoshino et al. [7] examined mercury cycling within the bay’s food web and reported that methylmercury cycling is strongly influenced by benthic microalgae. However, previous results have not identified the specific pathways through which methylmercury accumulates in fish.

Based on Hg monitoring conducted in 2011, the diss-MeHg concentration in Minamata Bay, where the dissolved oxygen (DO) concentration is relatively high (average value of 7.26 ± 1.11 mg/L), occasionally increased sharply to approximately 3–5-fold or more than the annual average [8]. Furthermore, statistical analyses of mercury monitoring data collected in Minamata Bay from 2014 to 2018 (2021 Hg monitoring results) showed that such phenomena were not season-dependent and were strongly influenced by the surrounding marine environment [9]. These findings suggest that environmental conditions may partially account for the continued existence of fish with high THg concentrations in the bay. Mercury methylation in marine environments generally occurs under reducing conditions characterized by low DO concentrations and low redox potential, such as in marine sediments where sulfate-reducing bacteria (SRB) harboring the hgcA, B gene are active [10,11,12]. However, MeHg can form even under oxic conditions with relatively high DO concentrations in seawater [13,14]. Research on mercury methylation under such conditions has traditionally been divided into studies of abiotic and biotic methylation. Abiotic methylation involves photochemical reactions and other non-biological processes that promote mercury methylation under oxic conditions [15,16]. However, these abiotic methylation mechanisms are generally insufficient to account for the majority of mercury methylation observed in marine environments [17]. In contrast, studies on biotic methylation have shown that anaerobic microenvironments may form within marine snow, which consists of suspended particulate matter (SPM) and other aggregates even in surface waters of the open ocean. Mercury methylation is therefore thought to occur primarily within these localized microenvironments [18,19]. Furthermore, SPM and its aggregates in the ocean and zooplankton interiors can create anoxic microhabitats suitable for anaerobic bacteria: living zooplankton themselves can serve as sites for methylmercury synthesis [20]. Moreover, recent research strongly suggests that the methylation of divalent mercury under oxic conditions does not involve hgcAB genes [21]. However, no unified mechanism has yet been established, and mercury methylation under oxic marine conditions remains poorly understood. Accordingly, it is necessary to determine whether the sudden increases in dissolved methylmercury concentrations in the surface waters of Minamata Bay, where DO levels are relatively high, are attributable to the reactions described above. A better understanding of mercury cycling in Minamata Bay is therefore essential. Based on 2021 Hg monitoring results and other relevant data, we experimentally investigated the conditions under which artificial mercury methylation could occur in seawater without substantially deviating from the current seawater quality conditions of Minamata Bay. We then prepared Minamata Bay seawater samples through membrane filters with different pore sizes and performed incubation experiments under conditions obtained from the incubation experiments. The results demonstrated that mercury methylation under oxic conditions in Minamata Bay was dependent on the membrane pore size used for seawater filtration.

## 2. Materials and Methods

### 2.1. Sampling Points

Minamata Bay covers an area of approximately 322 ha and has an average depth of 16.7 m (Kumamoto Prefecture, 1998). Seawater samples for the experiment were collected from a fixed sampling site located on the quay near the Minamata Bay Ferry Terminal.

### 2.2. Seawater Sample Collection and Pretreatment

Surface seawater from Minamata Bay (DO concentration: 6.97 ± 1.73 mg/L; redox potential: 153.11 ± 37.80 mV) was collected and used unfiltered in experiments designed to evaluate the influence of environmental factors on mercury methylation.

To investigate the relationship between mercury methylation and the particle-size fractions of SPM, surface seawater from Minamata Bay was filtered in advance using polycarbonate membrane filters with pore sizes of 0.20, 0.45, 0.8, and 3.0 µm; the resulting filtrates were used in the incubation experiments. To prevent cross-contamination during filtration, all glassware used in the filtration apparatus was cleaned with 10 mL of a strong acid mixture (HNO_3_:HClO_4_:H_2_SO_4_ = 1:1:3, *v*/*v*/*v*). The glassware was subsequently rinsed thoroughly with tap water and finally with distilled water before use.

**Figure 1:**
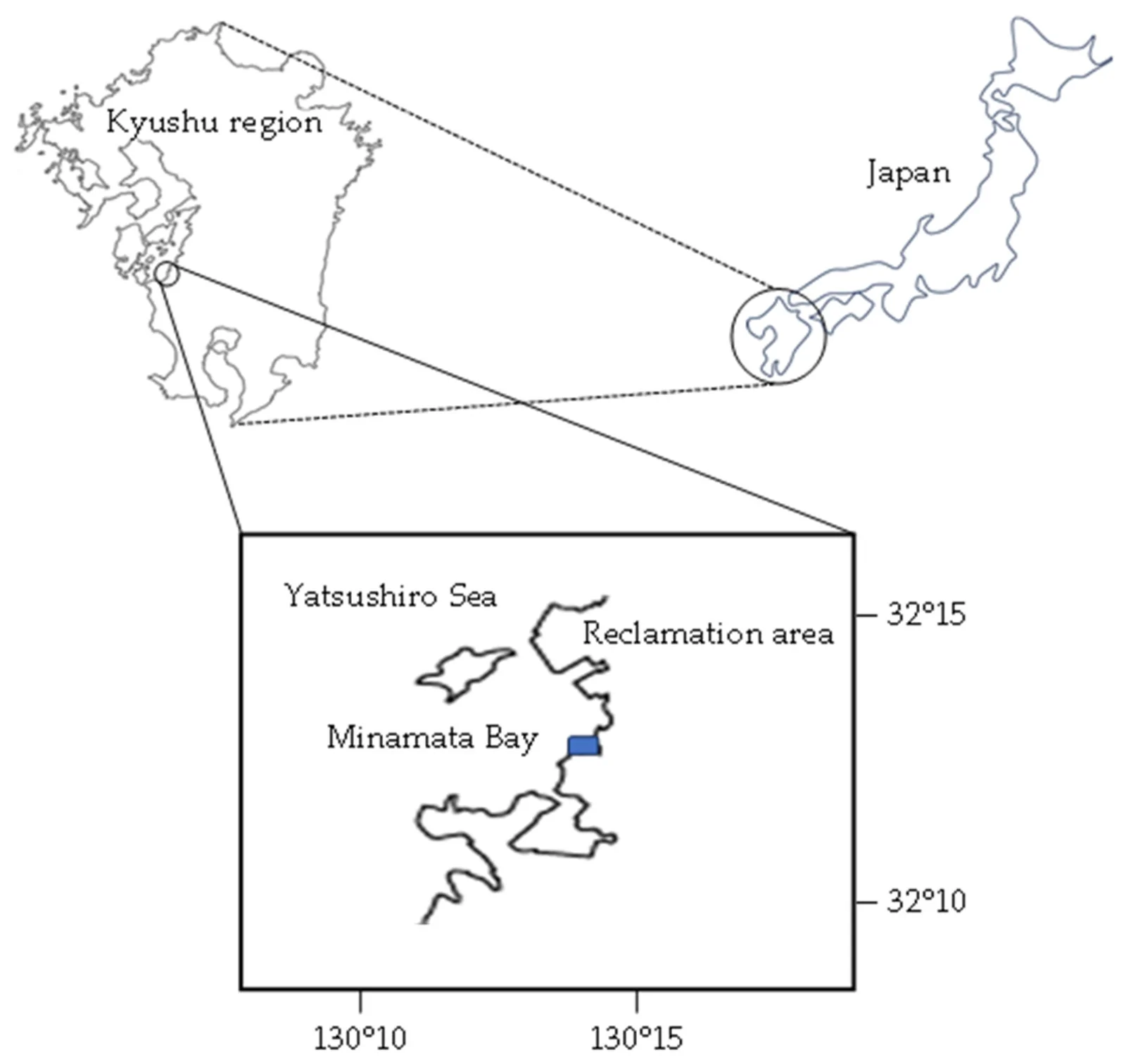
Sampling location within Minamata Bay. 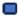: Seawater sampling point for incubation experiment in Minamata Bay. The seawater depth at the sampling point was from the surface to −2 m. There is no direct river flow into Minamata Bay.

Surface seawater samples were collected in polyethylene containers (20 L) at the Minamata Port wharf throughout the year (Figure 1). Prior to collection, the containers were cleaned using an ultrasonic cleaner (Aiwa Medical Industry Co., Ltd., AU508CB, Tokyo, Japan). Subsequently, 200 mL of a strong acid cleaning solution, prepared by mixing 2 mL of pure nitric acid (HNO_3_), 2 mL of pure perchloric acid (HClO_4_), and 10 mL of pure sulfuric acid (H_2_SO_4_) with ion-exchange water, was added to each container, which was then shaken thoroughly to clean the inner surfaces. The containers were then rinsed at least five times with ion-exchange water before use. After collection, the seawater samples were transported from Minamata Port to the laboratory within 20 min.

### 2.3. Incubation Experiment

#### 2.3.1. Setting Environmental Incubator (Incubator) and Incubation Period

The incubation experiment was conducted using seawater and two environmental incubators (EYELA FLI-2000, Tokyo, Japan) operated simultaneously under dark conditions. Because the recirculating air inside the incubators could potentially contain trace amounts of elemental mercury released during the incubation experiments, measures were taken to prevent contamination and minimize experimental error. To achieve this, special activated carbon filters (UF-PF6-1000, UES Co., Ltd., Osaka, Japan) were installed over all ventilation openings of the incubators to remove trace elemental mercury and airborne particulate matter from the circulating air.

**Figure 2:**
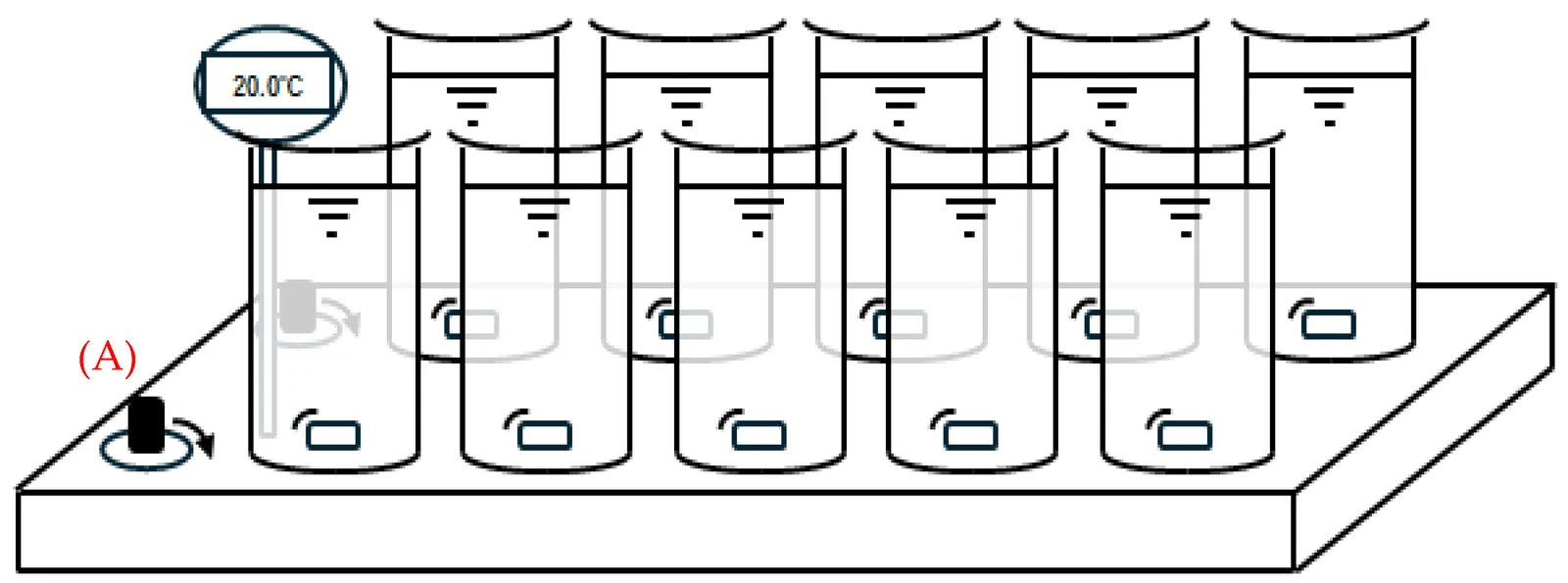
Incubation experiment conducted in the incubation tank showing the experimental state of the incubation experiment: incubation was conducted in the dark, and the temperature and humidity were adjusted to remain at constant conditions in the incubation tank. Turning knob (A) causes the stirrer inside the beaker to rotate.

Ten 500 mL aliquots of seawater were placed in individual tall glass beakers, which were then placed inside the incubator chambers. Before use, all beakers were cleaned with 5 mL of a strong acid mixture (HNO_3_:HClO_4_:H_2_SO_4_ = 1:1:3, *v*/*v*/*v*), thoroughly rinsed with tap water, and finally rinsed with distilled water. Incubation was performed under continuous stirring using magnetic stirrers while environmental parameters, such as seawater temperature, were systematically varied (Figure 2). The incubation period under dark conditions was initially set to 48 h; however, the duration was modified when necessary according to the measured diss-MeHg concentrations.

#### 2.3.2. Experimental Conditions for Incubation Experiments

We selected the environmental factors used in the incubation experiment based on the 2021 Hg monitoring results (using samples collected between 2014 and 2018) from Minamata Bay [9]. We previously demonstrated that seawater temperature, salinity, and DOC concentration significantly influence the production of diss-MeHg in Minamata Bay. This information was demonstrated through multiple regression analysis (explanatory variables: seawater temperature and DOC; response variable: diss-MeHg concentration; R^2^ = 0.69; explanatory variables: salinity and DOC; response variable: diss-MeHg concentration; R^2^ = 0.73). Therefore, these parameters were used as environmental variables in our current incubation experiment. The incubation conditions were set according to the 5-year average values reported in the 2021 Hg monitoring results, supplemented by recent mercury monitoring data from Minamata Bay (2024) [3].

- Seawater temperature: 17–26 °C

The overall average seawater temperature reported in the 2021 Hg monitoring results was 21.3 ± 1.0 °C, whereas the annual average in Minamata Bay in 2024 was 23.8 ± 2.0 °C (maximum of 28.6 °C, minimum of 21.6 °C, and 15.0 °C in winter). The temperature was controlled automatically by a sensor installed in the incubator, set to a predefined target temperature inside the incubator tank. However, as the temperature inside the incubator tank could differ from the actual seawater temperature, the relationship between the incubator tank temperature and actual seawater temperature was confirmed in advance. The actual seawater temperature, as measured by a portable electronic thermometer, was used to adjust the incubator settings.

- Seawater salinity: 30.5–35.5‰

The overall average salinity from the 2021 Hg monitoring results was 31.5 ± 3.0‰, whereas the 2024 annual average for Minamata Bay was 33.1 ± 1.2‰ (maximum of 33.9‰, minimum of 25.6‰). To decrease the salinity, ion-exchanged water was added to dilute the seawater. In contrast, to increase salinity, sodium chloride (analytical grade NaCl; Kanto Chemical Co., Inc., Tokyo, Japan), preheated at 600 °C for 3 h, was added and mixed thoroughly according to the target concentration.

- DOC concentration: 1, 3, and 5 mg/L

The average DOC concentration of Minamata Bay from the 2021 Hg monitoring results [9] was 1.9 ± 0.7 mg/L. To reflect the typical water quality conditions in Minamata Bay and obtain experimental results that more closely align with reality, the DOC addition concentrations were set at 1, 3, and 5 mg/L based on the average DOC concentration mentioned above. Dissolved organic matter (DOM), which generally exists as a source of DOC in the sea, is a collective term for dissolved organic matter consisting of the remains of marine microorganisms, exudates from phytoplankton, substances leached from sediments, and humic substances, such as humic and fulvic acid, from rivers [22]. In addition, a previous report indicates that fulvic acid in river water, which strongly influences the ocean environment, accounts for approximately 40% of the DOM dissolved in river water [23]. To optimize the incubation conditions, DOM containing fulvic acid was extracted from Minamata Bay seawater for potential use as the primary DOC source. However, humic and fulvic acids have a wide molecular weight range from less than 500 to more than 300,000, and an average molecular weight of approximately 20,000 to 30,000; therefore, they are not homogeneous substances [24]. Furthermore, the selective extraction of fulvic acid from seawater is technically complex, requiring careful pH adjustment, stepwise adsorption onto resin, and precise methodological control [25]. Moreover, because the composition and concentration of DOM fluctuate seasonally [26], obtaining a consistent and homogeneous supply is difficult. Other sources of DOM in the ocean, besides the humic substances mentioned above, include saccharides, such as glucose, and amino acids, which are supplied as photosynthetic products of phytoplankton that widely inhabit the ocean, including coastal areas, or during plankton biodegradation [27,28]. The objective of this incubation experiment was to obtain basic results on mercury methylation under oxic conditions in seawater. Therefore, although its composition differs considerably from that of natural marine DOM, glucose (SigmaUltra, 99.5% GC; Sigma-Aldrich Chemical Sciences Company, St. Louis, MO, USA), a homogeneous, low-molecular-weight organic compound widely used in marine microbial culture studies, was selected experimentally as the DOC source [29]. The amount of glucose added was calculated based on the DOC concentration of the seawater sample, the volume of seawater required for the incubation experiment, and the target DOC concentration.

- Diss-THg concentration in the experimental seawater

**Figure 3:**
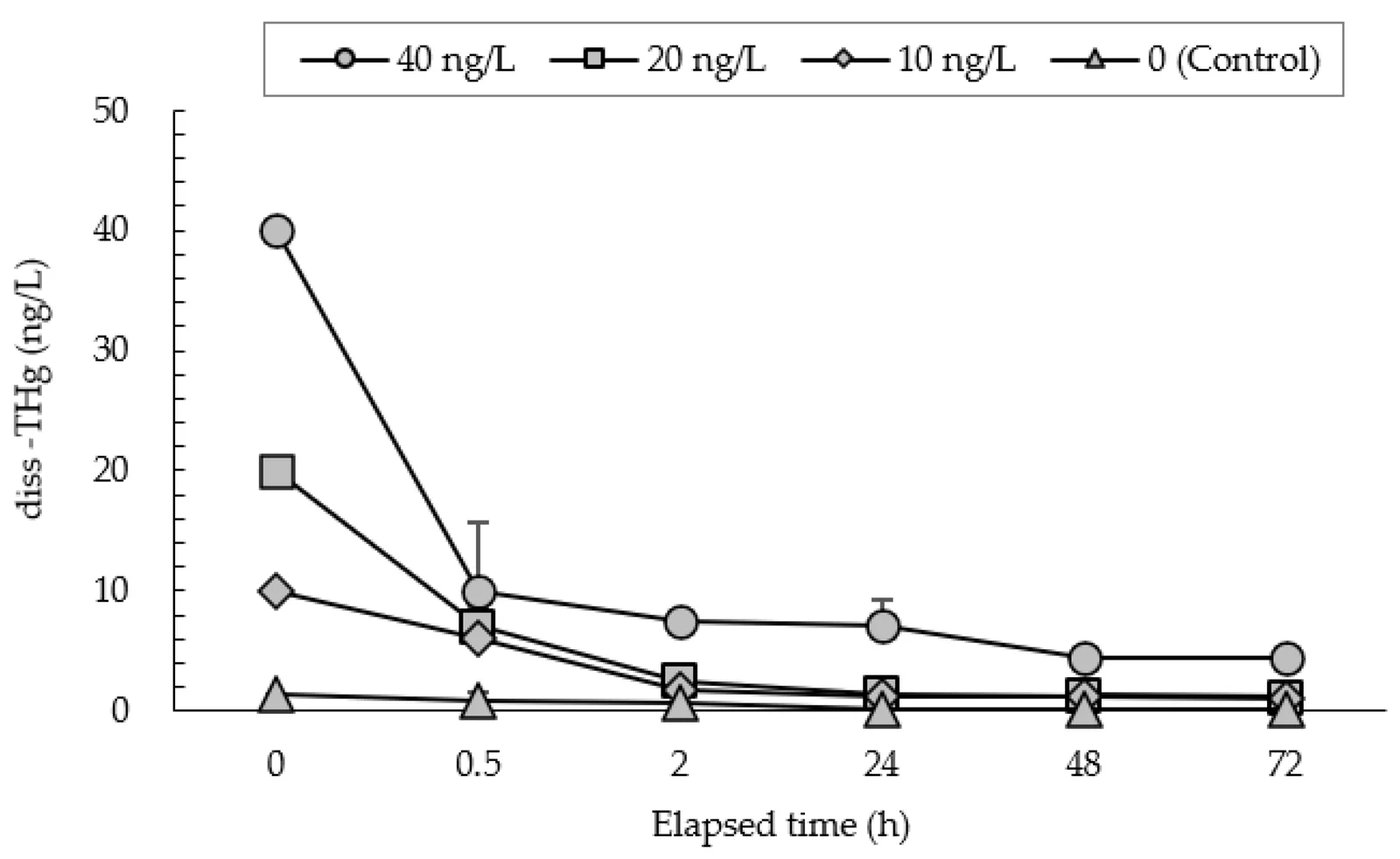
Time-dependent changes in the diss-THg concentration after adding HgCl_2_ (mercury equivalent: 10, 20, and 40 ng/L) to Minamata Bay seawater.

During the incubation experiment, we predicted that the concentration of divalent mercury in seawater would decrease because elemental mercury produced through marine microbial activity and other processes could volatilize into the atmosphere. Therefore, a preliminary experiment was conducted in which HgCl_2_ (analytical grade; Kanto Chemical Co., Inc., Tokyo, Japan) was added stepwise (equivalent to 10, 20, and 40 ng of mercury) to 1 L of Minamata Bay seawater filtered through a 0.8 µm polycarbonate membrane filter. This mixture was incubated continuously at 20 °C for 3 days, during which temporal changes in the dissolved mercury concentration were examined (Figure 3).

The results showed that the total mercury concentration in the seawater decreased to approximately 10% of the initial concentration within a few hours of incubation and continued to decline until reaching a stable level. Only the 40 ng addition case stabilized at approximately 5 ng after the initial decrease. Based on the 2021 mercury monitoring results, the annual average concentration of diss-THg in Minamata Bay was 0.38 ± 0.1 ng/L (maximum = 1.85 ng/L, minimum = 0.04 ng/L) [9]. Because unfiltered seawater was used in this incubation experiment, we considered the possibility that adsorption of mercury onto suspended organic matter and microbially mediated reduction of divalent mercury to elemental mercury via microorganisms would be greater than that observed in the preliminary experiments. Accordingly, 40 ng/L of mercury was added to the seawater in this experimental study based on the results of the preliminary experiments.

### 2.4. Mercury Analysis

The concentration of diss-MeHg in seawater was measured using the NIMD-A method (Akagi method) [30,31]. The standard protocol requires 1 L of seawater for diss-Hg extraction. However, 500 mL of seawater was used in the present study to evaluate time-series changes in diss-MeHg concentrations in the incubation experiments. For pretreatment, the sample seawater was filtered through a 0.45 µm membrane filter. Here, 5 mL of 0.01% dithizone-toluene solution (dz solution) was added to the filtered seawater and mixed to extract the diss-THg, including CH_3_Hg^+,^ into toluene as a dithizone complex. After extraction, 3 mL of 1 N NaOH was added to the toluene extraction, shaken, and the excess dithizone was removed into the aqueous phase. Subsequently, centrifugation was performed, and the lower aqueous phase was aspirated and removed. After removal, 2 mL of 0.15% Na_2_S solution was added, and the mixture was shaken to back-extract the mercury-dithizone complex from the toluene extraction into the aqueous phase. After back-extraction, the pH of the aqueous phase was adjusted to 3.0, and 0.05 mL of dz solution was added to this aqueous solution and mixed to extract the mercury from the aqueous phase into the toluene phase. After extraction, the purification procedure described above was performed again on the toluene extraction by adding 1 mL of 1 N NaOH to remove excess dithizone. The clear toluene phase obtained from this procedure was used as the sample for diss-MeHg analysis. The method detection limit (MDL) for MeHg was 0.01 ng/L, calculated from the standard deviation of six dithizone blanks. The reproducibility range of measurements was approximately 2–5%. In addition, to reconfirm the precision of this methodology, a recovery test was performed. A standard solution prepared by strong-acid digestion of DOLM2 dogfish muscle certified reference material (MeHg: 4.47 ± 0.32 mg/kg) was added to Minamata Bay seawater to obtain diss-MeHg concentrations of 0.02 and 0.04 ng/L, respectively. This recovery test was repeated four times. As a result, average recovery rates are 98.8 ± 0.6% using 0.02 ng/L diss-MeHg) and 99.1 ± 0.4% using 0.04 ng/L diss-MeHg) respectively. Also, this method has been validated as both reliable and accurate through an international intercomparison study using the ethylation method. The correlation coefficients from simple regression analysis between the Akagi and ethylation methods, applied to the same seawater samples, were 0.926 for diss-THg and 0.994 for diss-MeHg [32].

### 2.5. Measurement of the Physicochemical Properties of Seawater

The physicochemical properties of seawater were measured using the following equipment. The DO was measured with an integrated electrode (OE-570BA; TOA/DKK, Tokyo, Japan). The oxidation-reduction potential (ORP) was measured using an ORP electrode (PST-2729C; TOA/DKK, Tokyo, Japan). Salinity was determined using a YSI ProDSS 4-port digital sampling system (Xylem Japan, Kawasaki, Japan). The DOC concentrations were measured with a multi-N/C UV HS analyzer (Analytik Jena Japan, Yokohama, Japan). Seawater temperature was recorded using a digital thermometer (ST-9215; KENIS, Osaka, Japan).

### 2.6. Statistical Analysis

Differences among experimental groups were initially evaluated using one-way analysis of variance. When comparisons between groups were required, Welch’s *t*-test was applied because unequal variances were assumed. In addition, multiple comparisons were performed to identify significant differences among treatment groups. The Games–Howell post hoc test was selected because it is appropriate for datasets with unequal variances and sample sizes. The statistical significance level was set at *p* < 0.05, with values indicated by exact values or asterisks: *—*p* < 0.05. Statistical analyses were performed using diss-MeHg concentrations exceeding 0.09 ng/L obtained from repeated incubation experiments. Statistical analyses were conducted using IBM SPSS Statistics version 24 (IBM Corporation, Armonk, NY, USA) and Microsoft Excel 2019 (Microsoft Corporation, Washington, DC, USA).

### 2.7. Standard Environmental Monitoring Protocols and Reporting Guidelines

This study was in accordance with the Standard Guidelines for Environmental Monitoring of Chemicals established by the Ministry of the Environment, Japan [33].

## 3. Results and Discussion

### 3.1. Evaluation of Measured Data and Seawater Property Changes During Incubation

We obtained a total of 310 analytical data points from the incubation experiments using the diss-MeHg concentration as the dependent variable and DOC concentration, seawater temperature, and salinity as independent variables. According to the 2021 mercury monitoring results reported by Matsuyama et al. [9], excluding the 2017 dataset, during which diss-MeHg concentrations increased markedly, the average diss-MeHg concentration over the 4-year period from 2014 to 2018 was 0.03 ± 0.02 ng/L (*n* = 291).

Based on these results, the threshold value used to identify elevated diss-MeHg concentrations was calculated as the mean concentration (0.03 ng/L) plus three times the standard deviation (0.02 ng/L). Accordingly, only analytical results showing concentrations of 0.09 ng/L or higher were evaluated in this study. Table 1 and Table 2 summarize all data with diss-MeHg concentrations ≥ 0.09 ng/L. Notably, in experimental cases where the diss-MeHg concentration was ≥0.09 ng/L, no significant fluctuations in dissolved oxygen (DO) or oxidation–reduction potential (ORP) were observed during the incubation period (Figures S1 and S2). These results indicate that mercury methylation occurred without substantial changes in the bulk oxic conditions of the seawater during incubation.

### 3.2. Effect of DOC in Seawater on MeHg Production Process

Generally, inorganic mercury in seawater is known to undergo methylation through both biological and abiotic processes that occur within the water column [15,34]. Previous studies have reported that the production of diss-MeHg in the ocean is strongly positively correlated with the decomposition of particulate organic carbon [35]. The decomposition and cycling of organic matter associated with plankton play a critical role in MeHg generation in marine environments [36]. Figure S3. shows the percentage of samples with diss-MeHg concentrations ≥ 0.09 ng/L and <0.09 ng/L, classified by DOC concentration for all analytical data included in this study.

**Figure 4:**
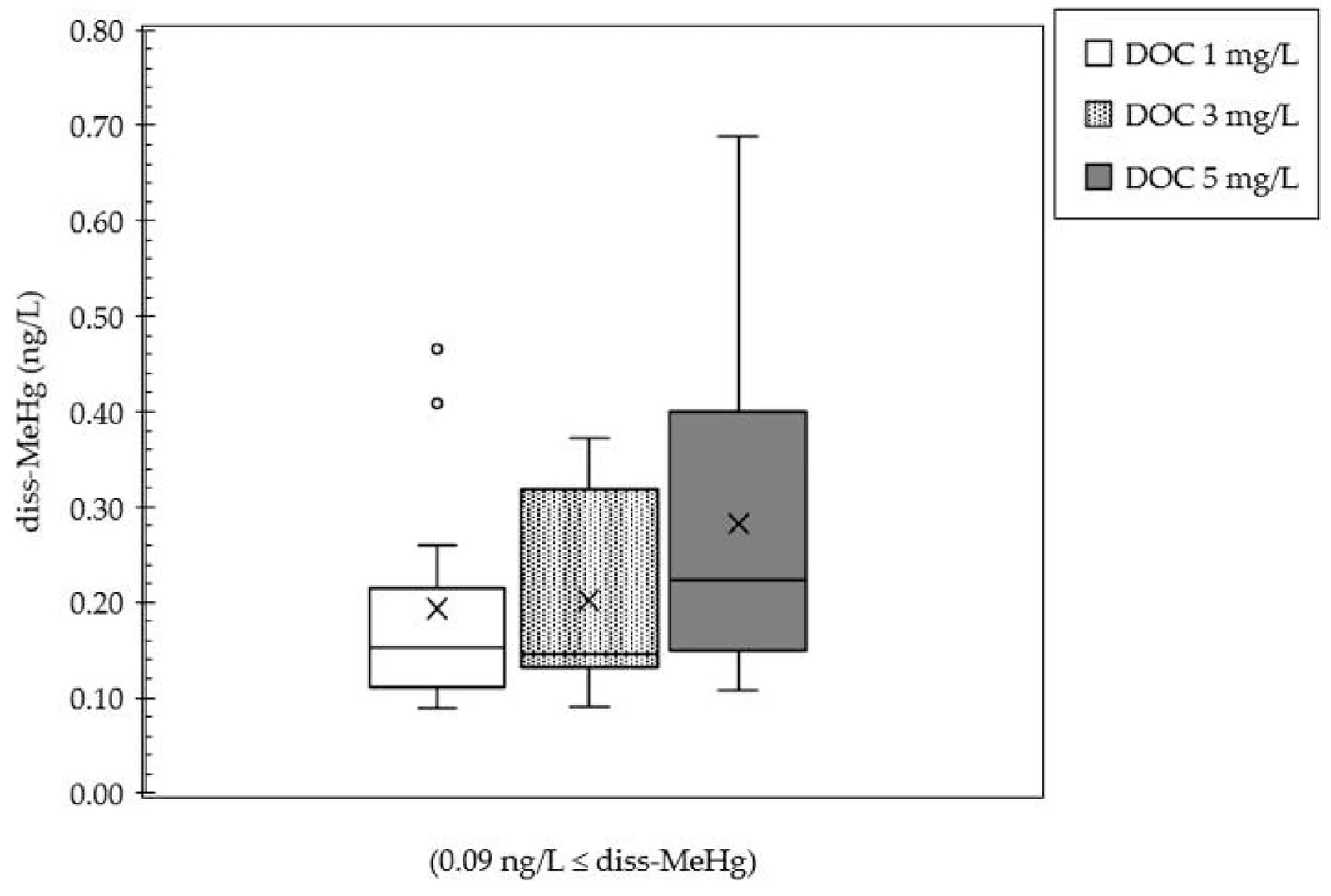
Distribution of diss-MeHg concentrations across varying DOC concentrations using only the analytical data showing diss-MeHg levels of 0.09 ng/L or higher from the 310 analytical data points. Means of symbols in the figure are as follows. ○: outlier, ×: average value, 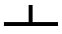: minimum value, 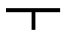: maximum value, horizontal line (―) in the box: median.

As shown in Figure S3, the proportion of samples with diss-MeHg concentrations ≥ 0.09 ng/L varied with DOC concentration. Overall, the average percentage of samples with diss-MeHg concentrations ≥ 0.09 ng/L was 21.8 ± 7.1%. However, when the data were grouped according to DOC concentration, the proportion of samples with diss-MeHg concentrations ≥ 0.09 ng/L, it was recognized that a tendency increased as DOC concentration increased (Figure 4). These findings indicate that the possibility of elevated DOC concentrations promote diss-MeHg production under the experimental conditions used in this study.

However, the 2021 Hg monitoring results showed an inverse trend, with diss-MeHg concentrations tending to decrease as the DOC concentrations increased. This discrepancy has not been thoroughly investigated, and the underlying mechanisms remain unclear. One possible explanation is the difference in the characteristics of dissolved organic matter (DOM) present in seawater. Large amounts of DOM exist in the ocean and serve as a source of DOC. DOM in seawater is generally defined as dissolved organic matter that passes through a 0.2–1 µm pore size filter [37]. DOM comprises labile dissolved organic matter (LDOM), semi-labile dissolved organic matter (SLDOM), and refractory dissolved organic matter (RDOM), which differ in their susceptibility to microbial degradation [38]. Rodriguez [17] suggested that seasonal variations in seawater components, such as particulate organic carbon (POC), may be due to differences between the experimental results reported by Lehnherr et al. [34] and field observation results. Furthermore, Hino et al. [26] reported that the amount of SLDOM derived from phytoplankton in Suruga Bay seawater has significant seasonal fluctuations. Therefore, the inverse relationship observed in the 2021 mercury monitoring data suggests seawater microbial activity arising from variations in DOM quality. Even when high DOC concentrations were measured instrumentally, the relative proportions of LDOM and RDOM in Minamata Bay seawater may have varied depending on the sampling period. In contrast, under the controlled incubation conditions of the present study, diss-MeHg concentrations increased as DOC concentration increased.

This difference may be attributed to the contrasting properties of natural DOM and the experimentally added DOC source. Glucose, which was used as the DOC source in this study, is a readily biodegradable low-molecular-weight compound and is naturally released by many phytoplankton species as a photosynthetic product. Diss-MeHg in seawater is generally produced by microorganisms, such as SRB, in seawater through biochemical reactions that methylate Hg^2+^ under anaerobic conditions [39,40].

As this study was conducted under oxic conditions with relatively high DO concentrations in seawater, the incubation experiment results reflect aerobic rather than anaerobic conditions. As mentioned in the introduction, mercury methylation under oxic conditions mainly occurs in anoxic microenvironments formed within SPM or SPM aggregates in seawater. However, the present study also considered additional potential methylation pathways operating under bulk oxic conditions. LDOM rapidly binds with divalent mercury in seawater, allowing it to be readily consumed by microorganisms [41]. Furthermore, the presence of DOM may alter cell membrane properties, potentially promoting the initial stage of divalent mercury uptake [41].

Sukekawa et al. [42], in laboratory experiments using marine bacteria and glucose, reported that the DOC concentration of the added glucose decreased between 24 and 84 h after addition, but subsequently increased over time, indicating that bacteria-derived DOM was released into the water. These results suggest that LDOM, such as glucose, is readily utilized by microorganisms; its addition leads to the production of new DOM. Tada et al. [43] conducted a microbiological survey of seawater in Minamata Bay and, through metagenomic analysis of microorganisms, identified the presence of mercury methylation genes (hgcA, hgcB) necessary for the expression of mercury methylation in seawater. These findings indicate that microorganisms with the genetic potential for mercury methylation are present in the bay. Therefore, when bioavailable Hg^2+^ is present, both abiotic and biologically mediated methylation pathways may contribute to MeHg production, even under oxic conditions. Consequently, the enhanced diss-MeHg production observed in the present study was likely influenced by the addition of glucose as a readily available monosaccharide carbon source under oxic conditions. Furthermore, Hg^2+^ in natural seawater is generally associated with organic matter, similar substances, or as chloride complexes [8]. To methylate Hg^2+^ in the ocean, this Hg^2+^ must first dissociate from such organic matter and exist as a free Hg^2+^. In contrast, since Hg^2+^ was added in the form of HgCl_2_ at the start of this incubation experiment, the system contained an abundance of reactive Hg^2+^. Therefore, both the characteristics of the DOC source and the chemical speciation of Hg^2+^ differed substantially from those in the natural marine environment.

As a result, the production of diss-MeHg in seawater, as observed in the incubation experiments of this study, cannot be regarded as an exact representation of mercury methylation under natural marine conditions, due to variations in the properties of DOM and the environmental conditions under the mercury methylation reaction. Therefore, the microbial activity observed in these experiments may not reflect that in typical marine environments; the process of dissolved methylmercury production may also differ accordingly. These differences make it difficult to directly compare the results of this experiment with the results of mercury monitoring surveys in Minamata Bay. However, based on previous studies by Sunderland et al. [35] and Cossa et al. [36], as well as our experimental results, carbon sources in the ocean can be easily decomposed and utilized by microorganisms; thus, microbial activity increases with higher DOC concentrations in seawater, and there is a possibility of mercury methylation promotion [35,36].

### 3.3. Relationship Between Time-Series Changes in Production of Diss-MeHg and DOC Concentration

**Figure 5:**
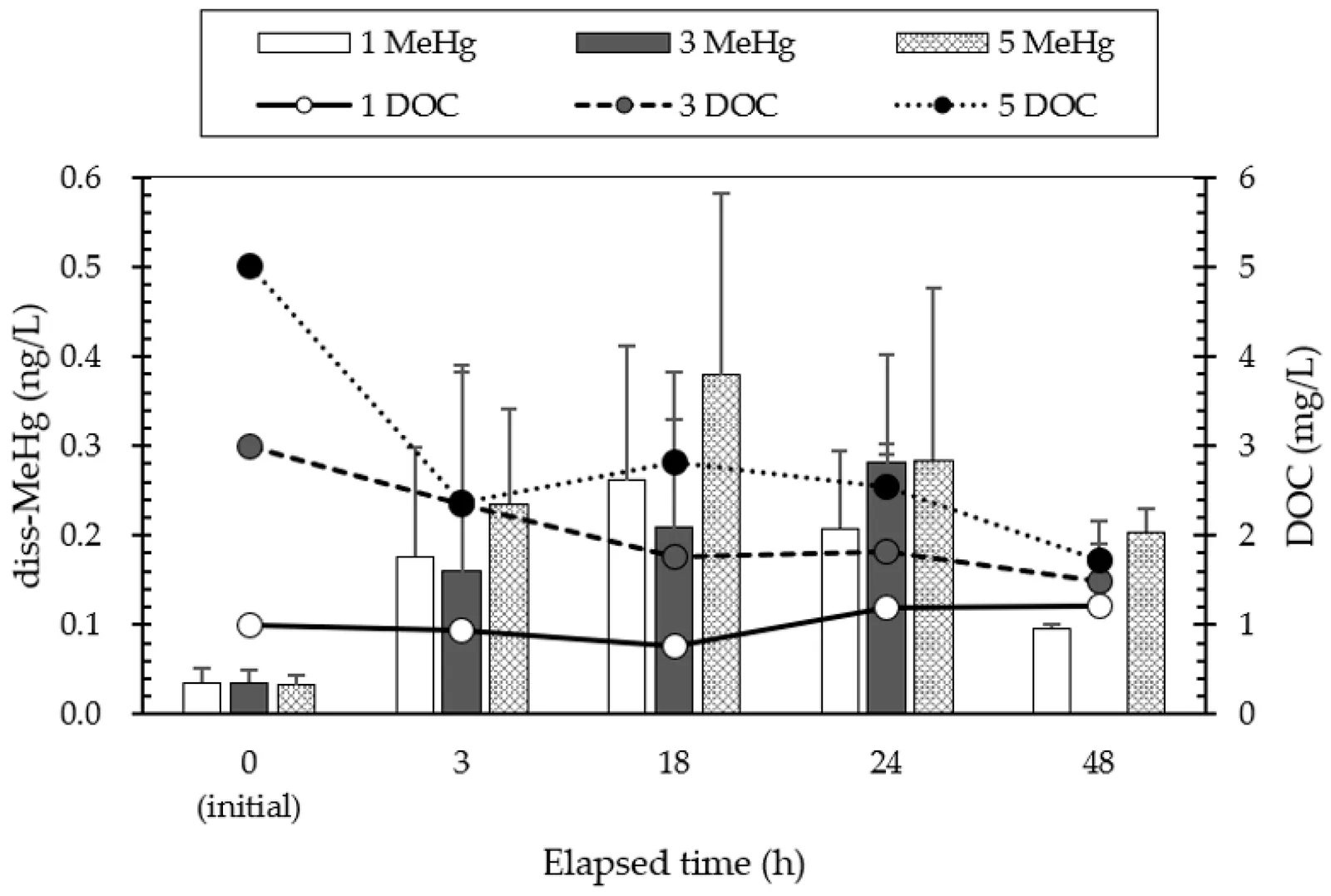
Temporal variation in the diss-MeHg and DOC concentrations during the incubation experiments.

Figure 5 shows the time-series changes in the diss-MeHg and DOC concentrations for samples (*n* = 69) in which the diss-MeHg exceeded 0.09 ng/L based on sample analyses obtained during the incubation experiment. As shown in Figure 5, at DOC concentrations of 3 and 5 mg/L, DOC decreased rapidly during the first 3–18 h after glucose addition, with a more pronounced decline observed at 5 mg/L than at 3 mg/L. In contrast, little change in DOC concentration was observed immediately after incubation at a DOC concentration of 1 mg/L.

Although the absolute diss-MeHg concentrations differed among DOC treatments, the temporal pattern of diss-MeHg production was generally similar across all DOC concentrations. Following the initial decline in DOC, diss-MeHg concentrations increased and subsequently remained elevated. Notably, diss-MeHg concentrations remained ≥ 0.09 ng/L for 48 h only in the 5 mg/L DOC treatment, suggesting that elevated DOC concentrations may sustain diss-MeHg production under the experimental conditions.

The rapid decrease in DOC observed during the early incubation period likely reflects microbial utilization of the added glucose, whereas the sustained diss-MeHg production at the highest DOC concentration suggests that continued carbon availability supports microbial processes associated with mercury methylation.

The relationship observed in this study is approximately consistent with the results shown in Figure 4 (Section 3.2), confirming a close relationship between DOC addition via glucose and diss-MeHg production.

### 3.4. Effect of Seawater Temperature on MeHg Production

**Figure 6:**
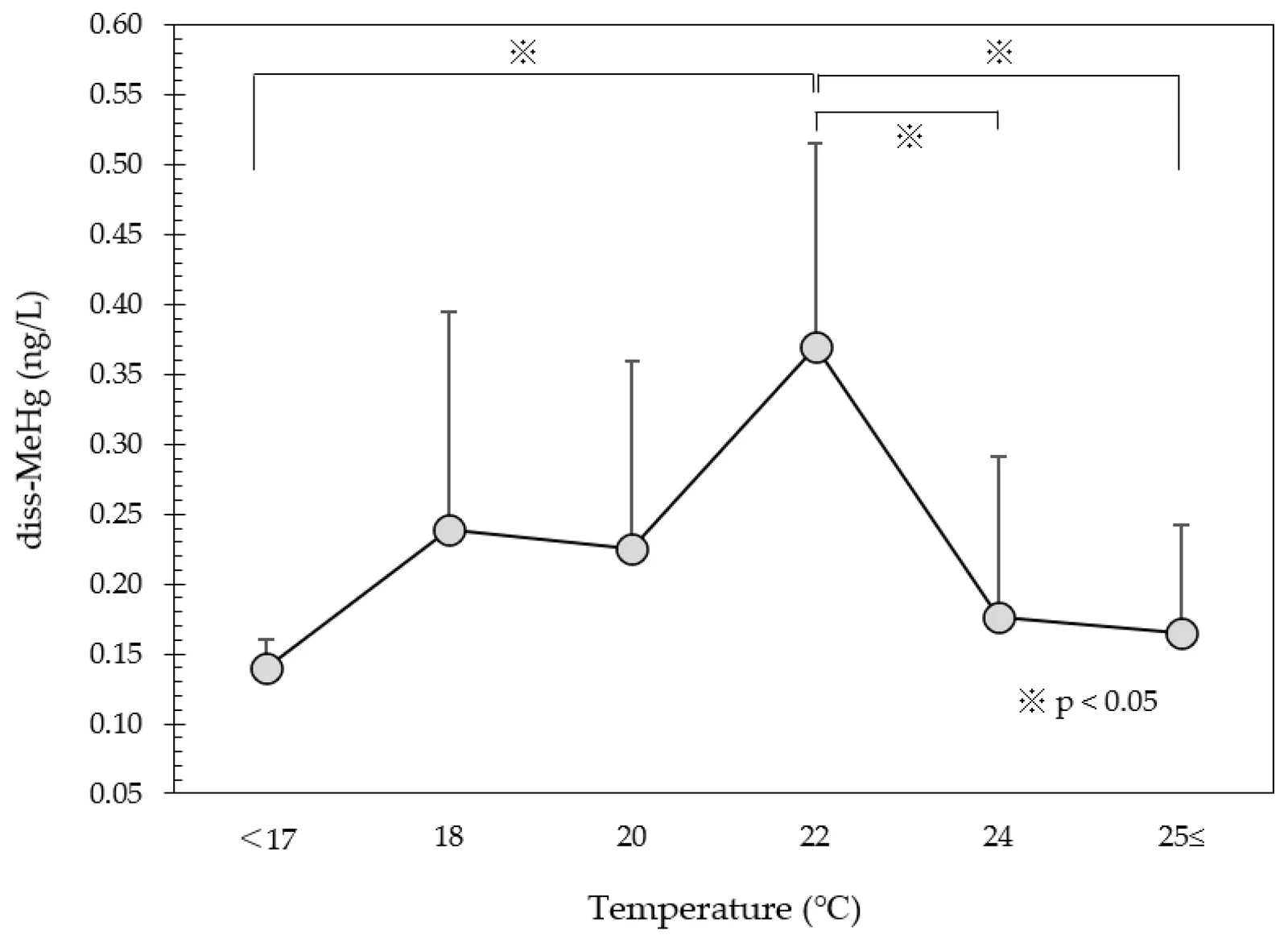
Relationship between the diss-MeHg concentration and seawater temperature.

Water temperature is a critical environmental factor that influences mercury methylation in aquatic environments [44]. Generally, the production of diss-MeHg in seawater is strongly affected by marine microbial activity. As seawater temperature increases, microbial activity intensifies, resulting in greater diss-MeHg production [14,45,46]. In contrast, under low-temperature conditions, the release of MeHg from sediments decreases by approximately 50–70% at 4 °C compared with that at 20 °C. This decrease has been ascribed to reduced metabolic activity and the growth rates of microorganisms at lower temperatures [47]. Moreover, the effect of water temperature on mercury methylation in aquatic environments varies depending on the characteristics of the water body and weather conditions [40]. Figure 6 illustrates the relationship between the diss-MeHg concentration and seawater temperature observed in this study. The 2021 Hg monitoring results showed a positive correlation between the diss-MeHg concentration and seawater temperature [9]. Specifically, as seawater temperature increased from approximately 19 to 20.5 °C, the diss-MeHg concentration also increased.

**Figure 7:**
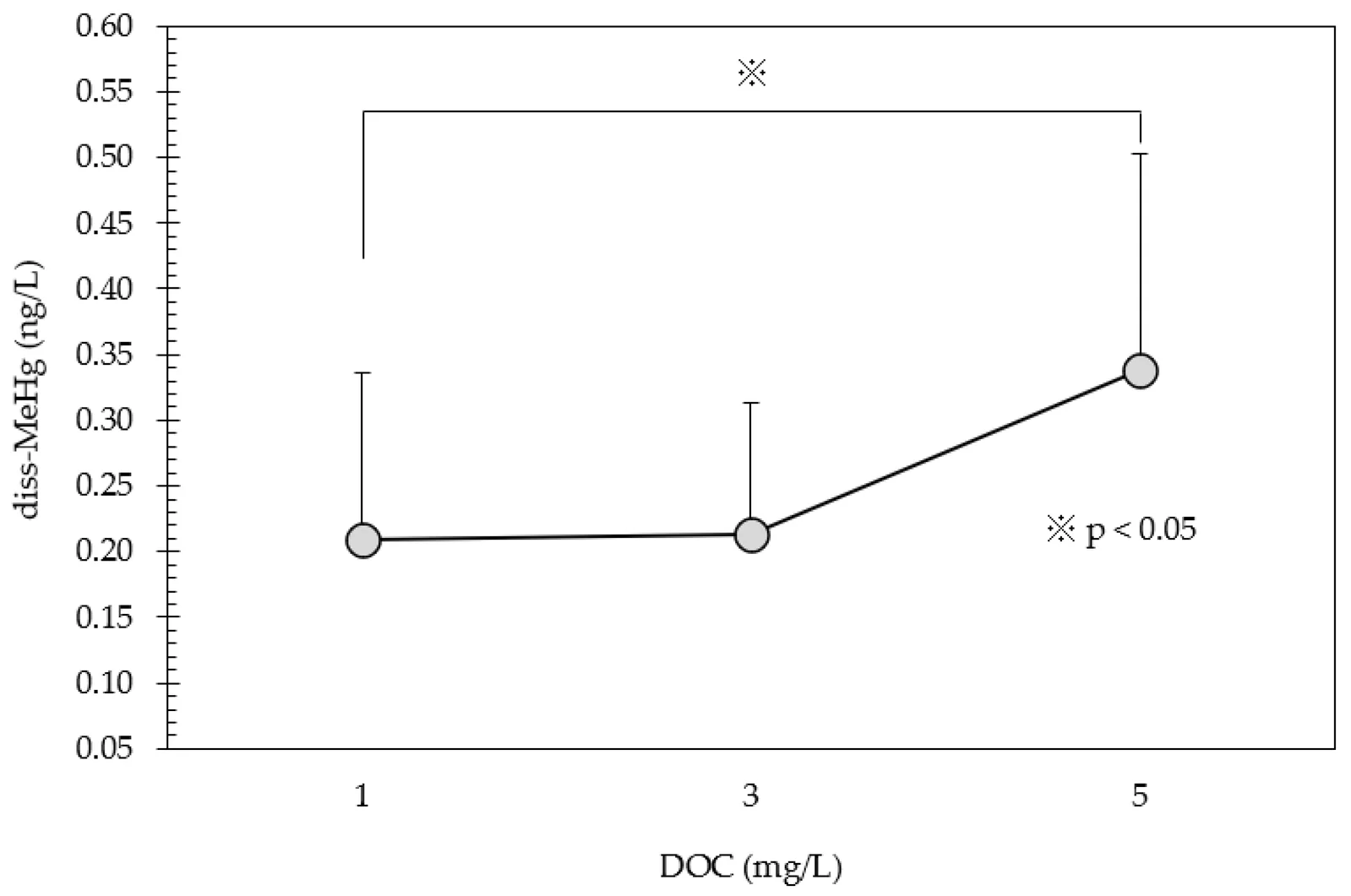
Relationship between the diss-MeHg concentration and added DOC concentrations at seawater temperatures between 18 and 22 °C.

In this incubation experiment, seawater with a salinity of 33.5 ± 1.6‰ was used, and incubation experiments were conducted at six different seawater temperatures. The results showed that the highest diss-MeHg concentration was observed at a seawater temperature of 22 °C. For the seawater temperature of 22 °C, which had the highest diss-MeHg concentration, there was no significant difference in the diss-MeHg concentration found between 18 and 22 °C based on the statistical results, whereas the diss-MeHg concentrations were significantly lower at temperatures outside this range. A seawater temperature of 22 °C, at which the diss-MeHg concentration peaked, closely matched the annual average surface seawater temperature of the Yatsushiro Sea (21.2 °C) where Minamata Bay is located (Kagoshima Prefecture, 2022). This finding suggests that the seawater temperature environment in Minamata Bay is relatively conducive to mercury methylation. Furthermore, to evaluate the effect of added DOC, the distribution of the diss-MeHg concentrations was categorized by DOC concentration using data from seawater temperatures between 18 and 22 °C. The results showed significant differences in the diss-MeHg concentration between DOC concentrations of 5 and 1 mg/L (Figure 7).

This result shows a trend similar to that shown in Figure 4 (Section 3.2), with a DOC concentration of 5 mg/L corresponding to the highest diss-MeHg concentration. Therefore, although DOC plays a key role in diss-MeHg production in seawater, statistical analysis indicated that the DOC concentration and seawater temperature are important factors that enhance the effect of DOC addition. Therefore, mercury methylation in Minamata Bay may be promoted within the natural range of seawater temperatures, depending on the prevailing environmental conditions.

### 3.5. Effect of Seawater Salinity on MeHg Production

**Figure 8:**
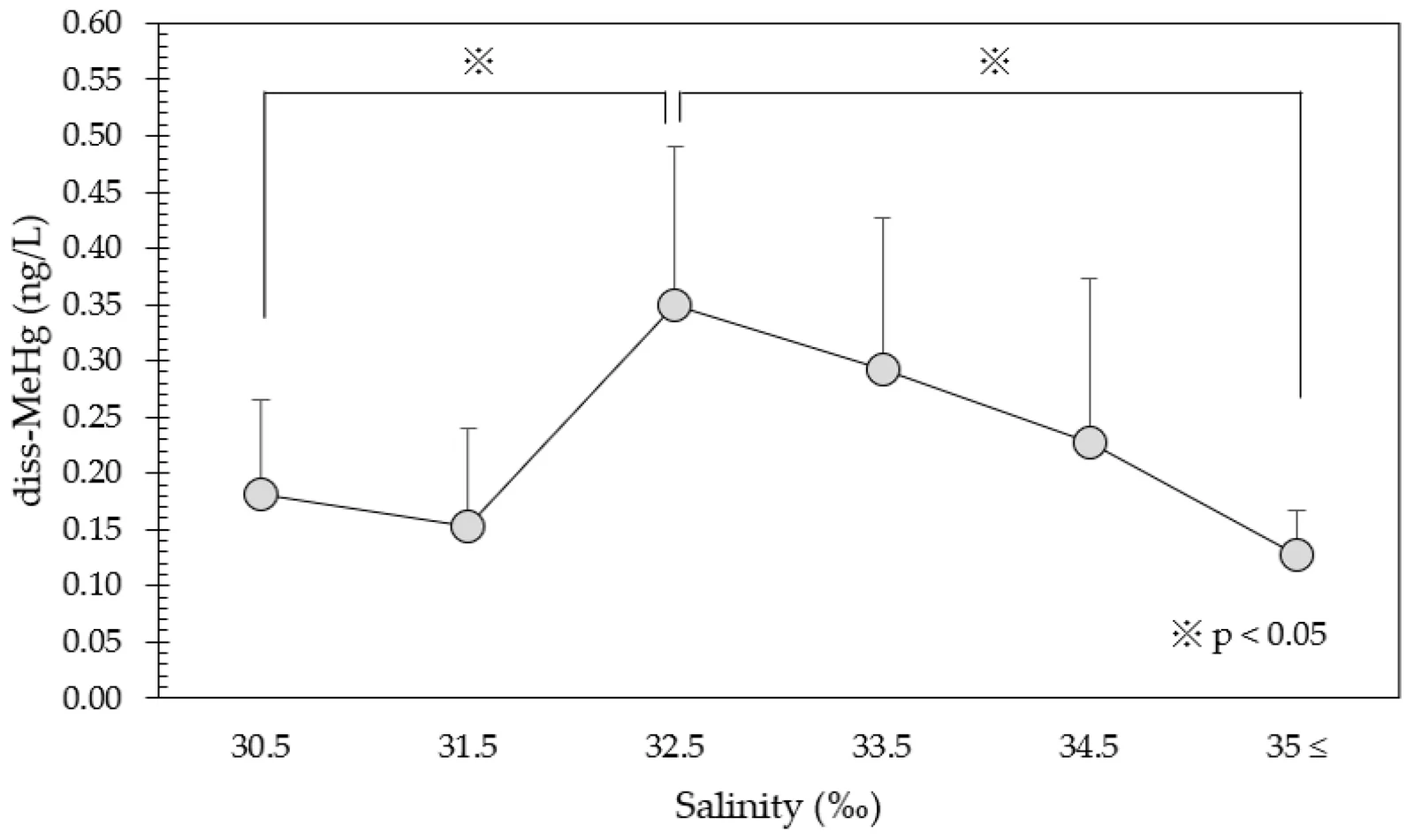
Relationship between diss-MeHg concentration and salinity.

The production of diss-MeHg in seawater is reportedly influenced by salinity [9,40,48]. Several studies have shown that mercury methylation decreases with increasing salinity [9,10,49]. Conversely, lower salinity conditions have been associated with enhanced mercury methylation [11,50]. Li et al. [51] reported that a salinity of 10‰ in seawater produced the highest levels of methylmercury in sediment via incubation experiments. However, some studies have reported no significant relationship between salinity and MeHg production in marine environments [52,53]. Collectively, these inconsistent findings indicate that the influence of salinity on mercury methylation remains incompletely understood. Figure 8 shows the relationship between the diss-MeHg concentration and salinity in seawater based on the results of our incubation experiments. The 2021 Hg monitoring results showed a negative correlation between the diss-MeHg concentration and salinity [9]. In contrast, the present study showed that diss-MeHg concentrations reached a maximum at a salinity of 32.5‰ and decreased as salinity either increased or decreased from this value. Furthermore, the 32.5‰ treatment exhibited the highest diss-MeHg concentration, although a statistically significant difference was observed only between the 32.5‰ treatment and salinities greater than 35‰. Increasingly, the salinity of 32.5‰ showed the highest diss-MeHg concentration; there was only a statistically significant difference observed between 32.5‰ and salinities greater than 35‰ and 30.5‰ treatment.

**Figure 9:**
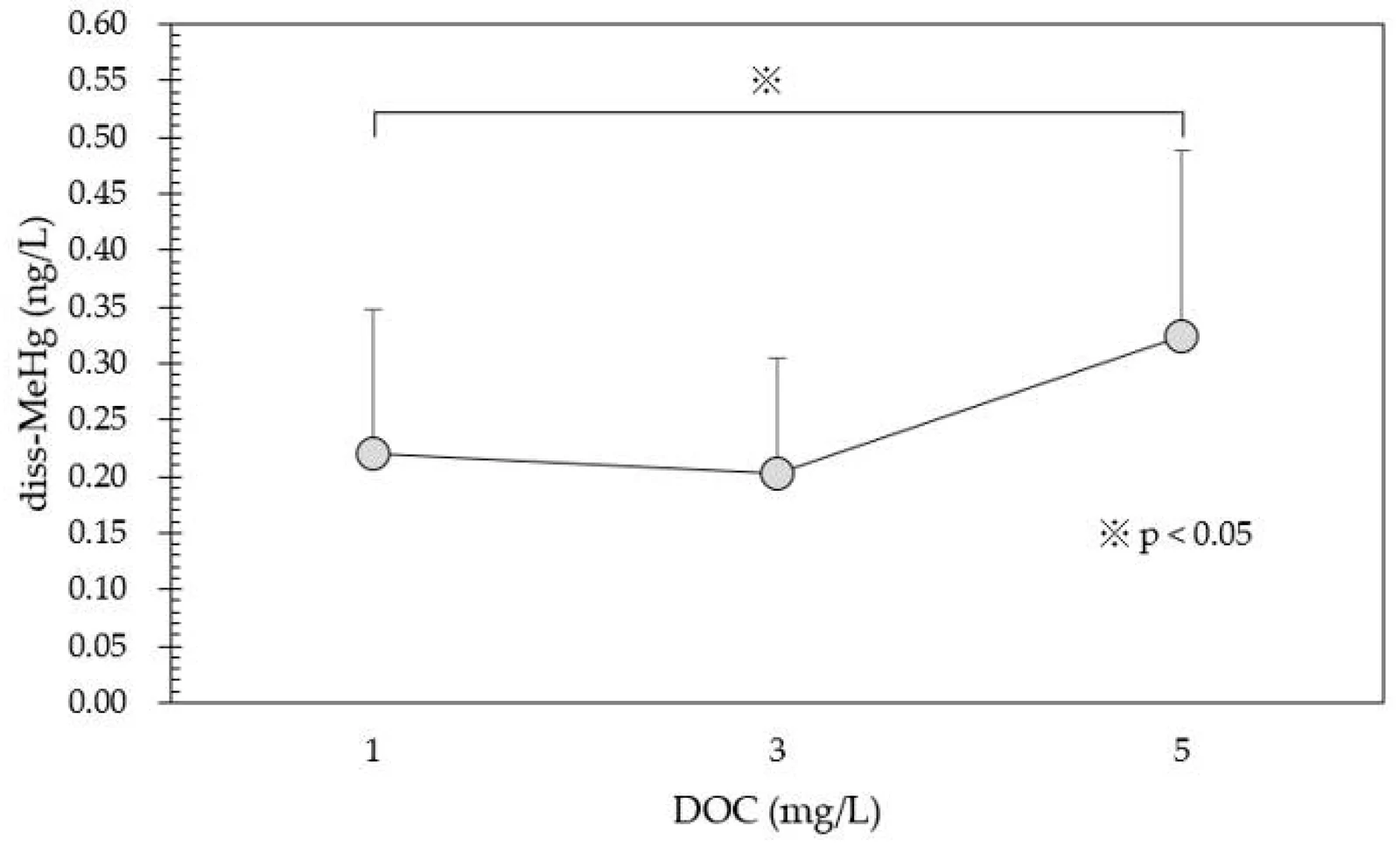
Relationship between the diss-MeHg concentration and DOC at a salinity of 31.5 to 34.5‰.

Figure 9 shows the distribution of diss-MeHg concentrations over a salinity range where there was no significant difference in the diss-MeHg concentration (31.5–34.5‰) based on DOC concentrations. As a result, there was a significant difference between 5 mg/L and 1 mg/L. The effect of seawater salinity shown in Figure 9 was similar to that of seawater temperature (Figure 7). In other words, diss-MeHg concentrations increased with increasing DOC concentration were observed. The focus of this study was mercury methylation under oxic conditions in normal marine environments. The salinity that had the highest diss-MeHg concentration (32.5‰) was similar to the surface salinity (33.1‰) of the Yatsushiro Sea [54]. Also, the average value of seawater salinity in Minamata Bay over the past 5-years is 31.54 ± 3.0‰ [9]. This finding suggests that mercury methylation under oxic conditions may be favored within the salinity range typically observed in the coastal waters surrounding Minamata Bay.

The focus of this study was mercury methylation under oxic conditions in normal marine environments. Based on our findings, mercury methylation in Minamata Bay may occur across a relatively wide range of seawater salinity conditions typical of the bay, provided that salinity does not exceed 35‰.

### 3.6. Influence of DOC on Optimized Salinity and Seawater Temperature Ranges for Diss-MeHg Production in Seawater

First, analytical data on diss-MeHg were extracted within optimized seawater temperature and salinity (18–22 °C, 31.5–34.5‰) from all of the data for evaluation (dissolved MeHg concentration of 0.09 ng/L or higher; *n* = 69); these data were categorized by the concentration of added DOC, with a summary of their characteristics (Table 3).

As listed in Table 3, among the samples with a DOC concentration of 5 mg/L that exceeded a diss-MeHg threshold of 0.09 ng/L (*n* = 31), 65% of the samples met the conditions for both optimal salinity and seawater temperature. For the other DOC concentrations (1 and 3 mg/L), approximately 50% of the samples satisfied these same conditions. Furthermore, the average diss-MeHg concentration at each DOC concentration was significantly higher at a DOC concentration of 5 mg/L (diss-MeHg = 0.36 ± 0.16 ng/L). Based on these results, even under the optimal seawater salinity and temperature conditions identified in the present incubation experiments, a DOC concentration of 5 mg/L could strongly induce mercury methylation in seawater, as described in Section 3.3, Section 3.4 and Section 3.5. Finally, based on the results presented in Section 3.2, Section 3.4 and Section 3.5, the conditions selected to artificially induce mercury methylation were a seawater temperature of 22 °C, salinity of 32.5‰, and DOC concentration of 5 mg/L.

### 3.7. Investigation of Mercury Methylation Induction Under Oxic Conditions Using Microfiltered Seawater

**Figure 10:**
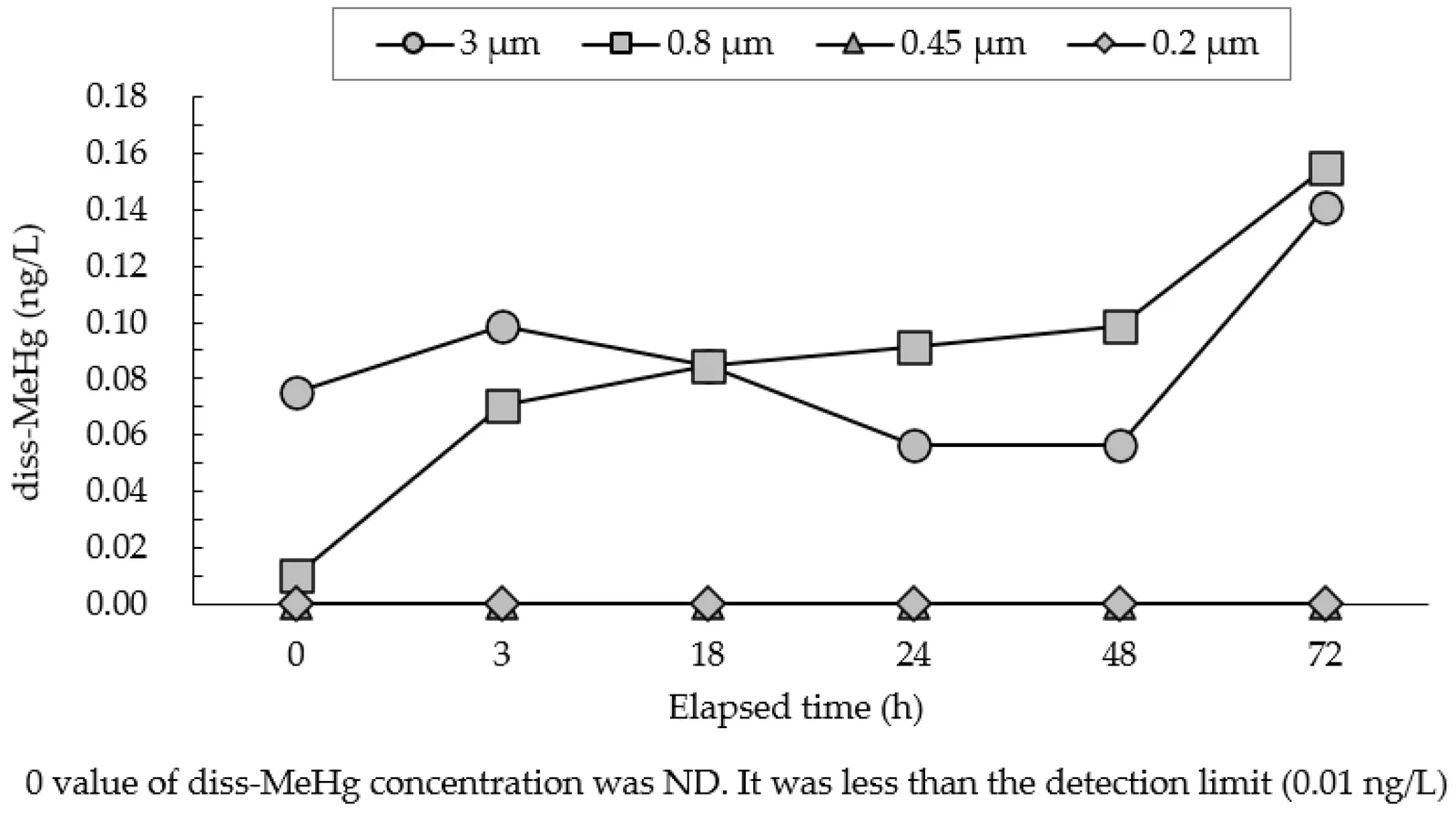
Time-series changes in the diss-MeHg concentration using incubation experiments with Minamata Bay seawater filtered by different pore sizes.

Based on the results obtained in this study, the artificial induction of mercury methylation in seawater required appropriate conditions of temperature, salinity, and DOC concentration. As mentioned above, mercury methylation in seawater under oxic conditions involves biological reaction processes in a reducing atmosphere in the microstructures formed within SPM and their aggregates, as well as non-biological processes involving chemical reactions (see Section 3.1). Furthermore, in both processes, DOM, DOC, and mineral components in seawater are involved in mercury methylation. Therefore, in this study, incubation experiments were conducted by applying the appropriate incubation conditions (see Section 3.6) to artificially induce mercury methylation in microfiltered seawater based on a stepwise method to investigate the characteristics of mercury methylation under oxic conditions. The basic setup for the incubation experiment was identical to the incubation experiment described above (see Section 2.2). The polycarbonate membrane filters used for microfiltration were set to four sizes: 3.0, 0.8, 0.45, and 0.20 µm. Based on the results of the incubation experiments (see Section 3.6), the incubation conditions were set to an incubation temperature of 22 °C, a salinity of 32.5‰, and a DOC concentration of 5 mg/L. Before starting the incubation experiment, the experiment was conducted with the addition of 40 ng/L of Hg (HgCl_2_). The DO concentration of seawater during the incubation period was 7.04 ± 0.28 mg/L. The pH of the seawater during the incubation period was 8.20 ± 0.07 (Figure 10).

**Figure 11:**
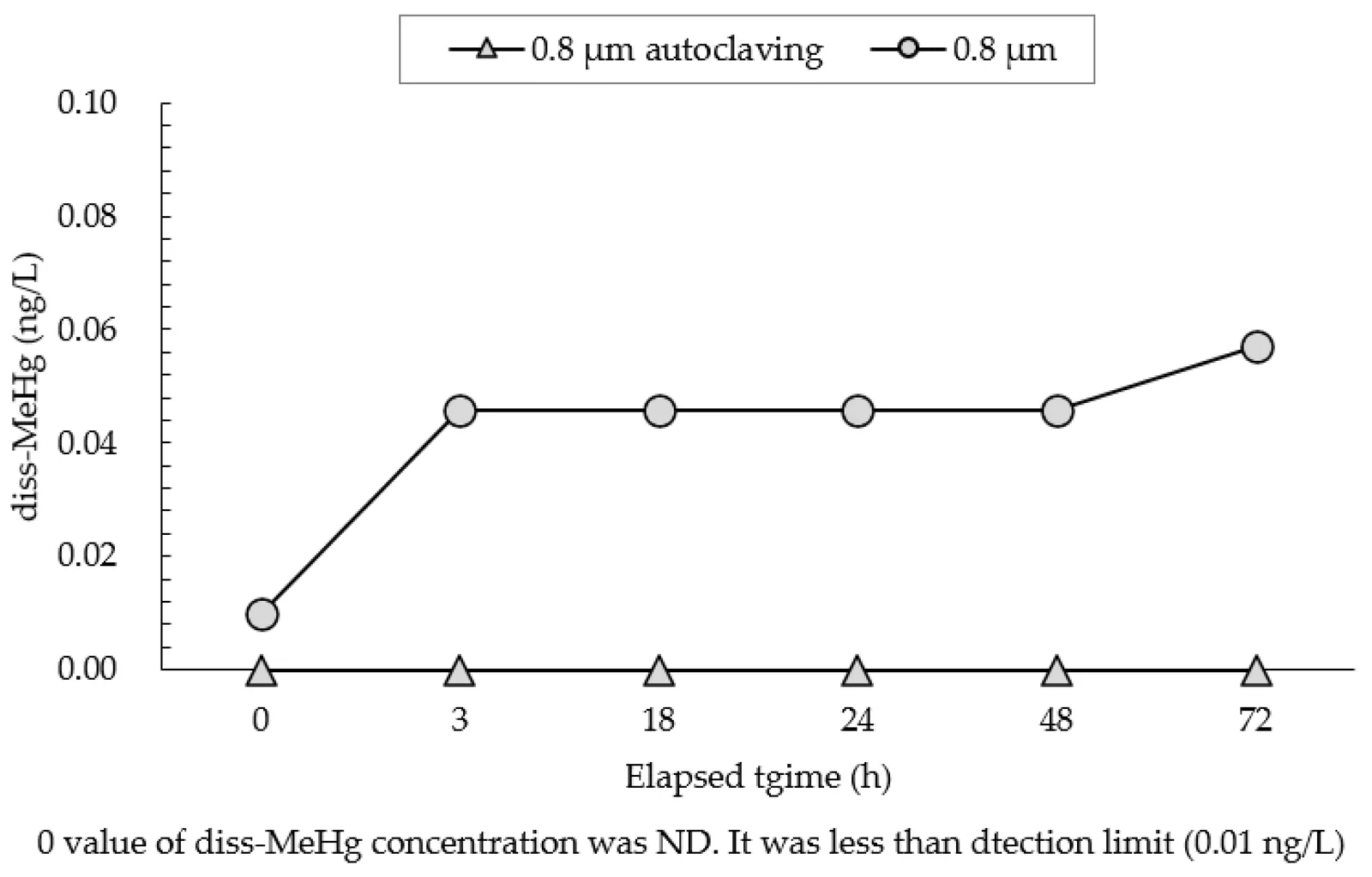
Time-series changes in diss-MeHg concentration using incubation experiments of Minamata Bay seawater filtered by 0.8 and 0.8 µm + autoclaving treatments.

Based on these results (Figure 10), diss-MeHg was less than the detection limit (0.01 ng/L) in seawater filtered with 0.20 and 0.45 µm membrane filters from the start, with no fluctuation in the diss-MeHg concentration during incubation. Therefore, mercury methylation was not detected in seawater filtered through the 0.20 and 0.45 µm membrane filters. In contrast, in seawater filtered with 0.8 and 3.0 µm membranes, the diss-MeHg concentration increased with each time-series change. Particularly, in seawater filtered with a 3.0 µm membrane filter, the diss-MeHg concentration was high immediately after the start of the experiment. This may be attributed to the rapid formation of diss-MeHg through reactions involving microbial residues and other fine organic particles present in seawater [34,55] or to the release of pre-existing diss-MeHg associated with SPM particles smaller than 3 µm. Thus, a clear difference in diss-MeHg production was observed at the 0.45 µm filtration boundary. Generally, filtration using a 0.45 µm pore size membrane filter can remove relatively large particles and some bacteria from seawater. A 0.8 µm membrane filter can remove larger bacterial clumps and suspended solids from seawater [56]. Therefore, although all visible relatively large particles and suspended solids were removed from seawater filtered using a 0.8 µm membrane filter, an increase in the diss-MeHg concentration was observed in the incubation experiments. Rodoriguez et al. [21] reported that the diss-MeHg concentration increased under oxic conditions at room temperature when DOM extracted from soil was added to Baltic Sea surface water in incubation experiments. Also, based on genome analysis of microorganisms in seawater, there is a completely different mercury methylation process that does not involve the hgcAB gene necessary for mercury methylation. Furthermore, Feng et al. [57] noted that mercury methylation also occurs in the aerobic soils of reservoirs. They conducted incubation experiments on mercury methylation under aerobic conditions using two strains of aerobic proteobacteria, using the global mercury cycle including terrestrial ecosystems. These findings suggest that aerobic microorganisms in marine environments may contribute to mercury methylation under oxic conditions. Therefore, microbial communities present in oxic seawater may play an important role in mercury methylation in Minamata Bay surface waters. To test this hypothesis, additional incubation experiments were performed using 0.8 µm filtered seawater under two conditions: (1) non-sterilized seawater and (2) seawater sterilized by autoclaving (121 °C, 1.2 atm, 30 min). For the incubation experiments, as the DO concentration of the sterilized sample decreased after autoclaving, it was set as a sterilized condition overnight. An average DO value for all seawater samples for the incubation experiments was 8.56 ± 0.56 mg/L (*n* = 12). An average value for the pH of all seawater samples for the incubation experiments was 8.20 ± 0.07 (*n* = 12) (Figure 11).

Based on these results (Figure 11), we did not detect diss-MeHg (detection limit of 0.01 ng/L) throughout the entire incubation period when the seawater samples were autoclaved. In contrast, when autoclaving was not performed, an increase in the diss-MeHg concentration was observed. Seawater characteristics change due to changes in inorganic mineral components and organic materials by autoclaving treatments [58]. Therefore, we cannot ignore the influences of altered minerals or organic materials due to autoclaving during mercury methylation. However, mercury methylation obtained in this study under oxic conditions was unlikely to be primarily associated with anaerobic bacteria, such as sulfate-reducing bacteria (SRB), inhabiting SPM and its aggregates, because these structures would have been substantially removed by filtration through the 0.8 µm membrane filter. Instead, the results suggest the involvement of a biologically mediated mercury methylation process performed by marine microorganisms capable of functioning under oxic conditions.

Furthermore, the results of this experiment may also explain the seasonal increase in the diss-MeHg concentration in Minamata Bay seawater, as evidenced by the 2021 mercury monitoring results, which do not match the distribution of the methylmercury concentration in SPM simultaneously present in the seawater [9]. In other words, diss-MeHg present in seawater originates from mercury methylation under anaerobic conditions within SPM and its aggregates, as well as from mercury methylation under oxic conditions through a different reaction pathway. Both pathways may occur simultaneously in marine environments, and their relative contributions may vary depending on environmental conditions, including organic matter availability, microbial communities, and mercury speciation. Therefore, the distribution of methylmercury in Minamata Bay may not match the diss-MeHg concentration in seawater and MeHg concentration in SPM.

### 3.8. Study Limitations

The incubation experiments used in this study were conducted within the normal range of natural conditions regarding seawater temperature and salinity observed in Minamata Bay. However, the experimental system differed from natural seawater conditions because glucose was used as the DOC source and HgCl_2_ was used as the Hg^2+^ source. Therefore, the chemical characteristics and reaction pathways may not fully represent those occurring in the natural marine environment. Furthermore, although this study demonstrated the possibility of mercury methylation by marine micro-organisms under aerobic conditions, a detailed study has not been conducted due to microbial phylogenetic analysis and their chemical characteristics, including abiotic reactions. Therefore, further research on oxic mercury methylation in seawater through metagenomic and microbial community analyses, investigation of reaction characteristics using saccharides other than glucose, and incubation experiments with the extraction and addition of fulvic acid from marine sediment.

## 4. Conclusions

In this study, to determine the environmental conditions under which mercury methylation can be artificially induced in Minamata Bay seawater using glucose as a DOC source and HgCl_2_ as an Hg^2+^ source, incubation experiments were conducted based on previous mercury monitoring results from Minamata Bay [8,9]. Consequently, although the incubation conditions qualitatively differ in comparison with natural marine conditions, seawater conditions facilitating artificial mercury methylation were elucidated. Next, the environmental conditions causing artificial mercury methylation were applied to seawater from Minamata Bay through stepwise microfiltration using membrane filters with different pore sizes, as well as artificial induction of mercury methylation under oxic conditions. The characteristics were examined based on the results of the incubation experiments. The environmental conditions adopted to promote mercury methylation in the incubation experiments were as follows: DOC concentration of 5 mg/L, seawater temperature of 22 °C, salinity of 32.5‰, and an added total mercury concentration of 40 ng/L (as HgCl_2_). The microfiltration membrane filters used with Minamata Bay seawater had pore sizes of 0.2, 0.45, 0.8, and 3.0 µm; the DO concentration of the used seawater sample was 7.04 ± 0.28 mg/L; and the continuous incubation period was 72 h. As a result, the diss-MeHg concentration of seawater filtered with 0.2 and 0.45 µm membrane filters was less than the detection limit (0.01 ng/L) from the start of the experiment, and there was no fluctuation in the diss-MeHg concentration during the incubation experiments. However, with the 0.8 and 3.0 µm filters, the production of diss-MeHg was recognized over time with fluctuations in the diss-MeHg concentrations. Therefore, to confirm whether this reaction originated from marine microorganisms, two types of seawater samples were prepared: one was filtered through a 0.8 µm filter and sterilized via autoclaving (121 °C, 30 min), whereas the control sample was subjected only to filtration. Consequently, no diss-MeHg was detected in the sterilized sample (less than 0.01 ng/L), indicating that mercury methylation may be largely influenced by marine microorganisms that can grow under oxic conditions. However, this study did not conduct detailed experimental investigations on mercury methylation under these oxic conditions, including microbial identification and the influences of mineral or organic material in seawater. Therefore, further investigations are necessary to clarify the mechanisms, ecological significance, and contribution of oxic mercury methylation pathways in marine environments.

## Figures and Tables

**Table 1:** Summary of all measurement data for diss-MeHg concentrations equal to or exceeding 0.09 ng/L.

DOC (mg/L)	All Data	Diss-MeHg ≥ 0.09 (ng/L)			
	*n*	*n*	Diss-MeHg (ng/L)	DO (mg/L)	ORP (mV)
			Average ± SD	Average ± SD	Average ± SD
1	128	26	0.19 ± 0.12	7.2 ± 1.3	210.0 ± 40.4
3	77	12	0.20 ± 0.10	7.0 ± 0.8	238.3 ± 50.9
5	105	31	0.28 ± 0.16	8.3 ± 1.6	193.3 ± 47.6

Abbreviations: DOC, dissolved organic carbon; DO, dissolved oxygen; ORP, oxidation–reduction potential; SD, standard deviation; *n*, number of replicates.

**Table 2:** Summary of all measurement data for diss-MeHg concentrations in varying temperature and salinity.

DOC (mg/L)	Diss-MeHg ≥ 0.09 (ng/L)					
	Temperature (Tp) °C			Salinity (Sal)‰		
	Range	*n*	Average ± SD	Range	*n*	Average ± SD
1	Tp < 17	2	16.05 ± 0.07	30 ≤ Sal < 31	4	30.73 ± 0.07
	17 ≤ Tp < 19	2	17.65 ± 0.49	31 ≤ Sal < 32	1	31.90 ± 0.00
	19 ≤ Tp < 21	9	20.00 ± 0.52	32 ≤ Sal < 33	2	32.00 ± 0.00
	21 ≤ Tp < 23	3	22.07 ± 0.15	33 ≤ Sal < 34	3	33.00 ± 0.00
	23 ≤ Tp < 25	5	23.68 ± 0.41	34 ≤ Sal < 35	12	34.77 ± 0.36
	25 ≤ Tp	5	25.76 ± 0.47	35 ≤ Sal	4	35.10 ± 0.06
3	Tp < 17	0		30 ≤ Sal < 31	1	30.23 ± 0.00
	17 ≤ Tp < 19	3	17.63 ± 0.71	31 ≤ Sal < 32	1	31.14 ± 0.00
	19 ≤ Tp < 21	4	20.55 ± 0.19	32 ≤ Sal < 33	4	32.12 ± 0.08
	21 ≤ Tp < 23	1	22.80 ± 0.00	33 ≤ Sal < 34	0	
	23 ≤ Tp < 25	1	23.50 ± 0.00	34 ≤ Sal < 35	4	34.92 ± 0.03
	25 ≤ Tp	3	25.80 ± 0.72	35 ≤ Sal	2	35.06 ± 0.02
5	Tp < 17	1	16.80 ± 0.00	30 ≤ Sal < 31	2	30.19 ± 0.01
	17 ≤ Tp < 19	5	17.86 ± 0.47	31 ≤ Sal < 32	0	
	19 ≤ Tp < 21	7	19.94 ± 0.53	32 ≤ Sal < 33	7	32.02 ± 0.04
	21 ≤ Tp < 23	10	22.09 ± 0.60	33 ≤ Sal < 34	5	33.00 ± 0.00
	23 ≤ Tp < 25	3	23.23 ± 0.21	34 ≤ Sal < 35	12	34.39 ± 0.48
	25 ≤ Tp	5	25.70 ± 0.37	35 ≤ Sal	5	35.13 ± 0.13

Abbreviations: DOC, dissolved organic carbon; SD, standard deviation.

**Table 3:** Effect of the stepwise addition of DOC at optimum salinity and temperature for diss-MeHg concentrations due to mercury methylation in the total analytical data.

Description	DOC Concentration (mg/L)		
	1	3	5
Total number of analytical data (*n* = 69)	26	12	31
The number of analytical data that both contain optimum salinity (31.5–34.5‰) and optimum temperature (18–22 °C) in the total *n*	10	6	20
Ratio of optimum salinity and temperature (B) to the amount of analytical data (A) B/A (%)	38	50	65
Average value of diss-MeHg concentration at optimum salinity and temperature (ng/L)	0.24	0.24	0.36
±SD	0.14	0.10	0.16
Multiple comparison test (Games-Howell) for 5 mg/L-DOC ※	※	※	

Abbreviations: DOC, dissolved organic carbon; diss-MeHg, dissolved methylmercury; SD, standard deviation; *n*, number of replicates. ※, the *p*-value is less than 0.05 for a DOC of 5 mg/L (*p* < 0.05). Multiple comparisons were adjusted using the Holm–Bonferroni method.

## Data Availability

The original contributions presented in this study are included in the article/Supplementary Material. Further inquiries can be directed to the corresponding author.

## Supplementary Materials

The following supporting information can be downloaded at: https://www.mdpi.com/article/10.3390/toxics14080717/s1, Figure S1: Fluctuations in DO during the incubation experiments; Figure S2: Fluctuations in ORP during the incubation experiments; Figure S3: Analytical sample ratio of ≥0.09 ng/L and <0.09 ng/L diss-MeHg with each DOC concentration based on the 310 analytical data points obtained from the incubation experiment.

## Abbreviations

The following abbreviations are used in this manuscript:

diss-MeHg	dissolved methylmercury
DOC	dissolved organic carbon
diss-Hg	dissolved mercury
diss-THg	dissolved total mercury
DO	dissolved oxygen
SRB	sulfate-reducing bacteria
