# Supplementary material for: Basic Study on Mercury Methylation Under Oxic Conditions: Incubation Experiments Using Seawater from Minamata Bay, Japan
Source: Toxics. 2026 Aug 13;14(8):717. doi: 10.3390/toxics14080717 (PMC13517714; doi:10.3390/toxics14080717)
Supplement: Supplementary file 1 [file toxics-14-00717-s001.zip › toxics-4430777-supplementary.pdf]

Supplemental Materials

Basic study on mercury methylation under oxic conditions:

incubation experiments using seawater from Minamata Bay, Japan

Figure of contents

Figure S1. Fluctuations in DO during the incubation experiments.

Figure S2. Fluctuations in ORP during the incubation experiments.

Figure S3. Analytical sample ratio of ≥ 0.09 ng/L and < 0.09 ng/L diss-MeHg with each DOC concentration based on the 310 analytical data points obtained from the incubation experiment.

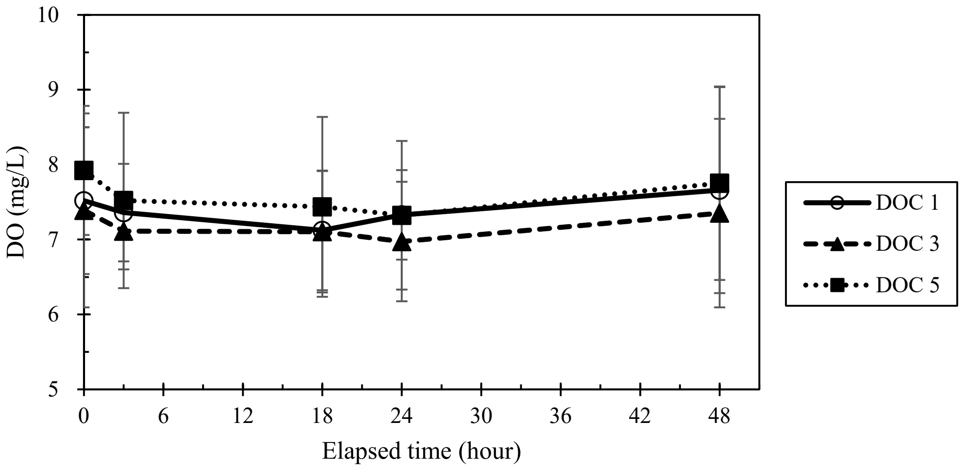

Figure S1. Fluctuations in DO during the incubation experiments.

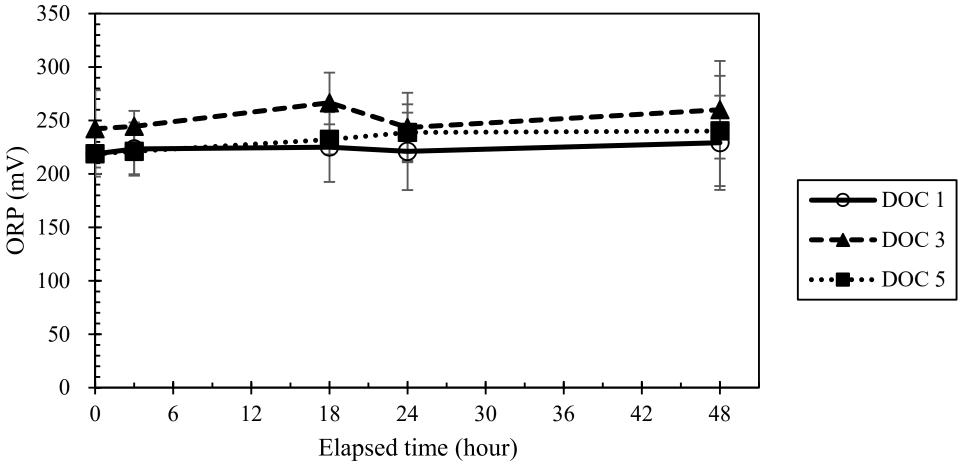

Figure S2. Fluctuations in ORP during the incubation experiments.

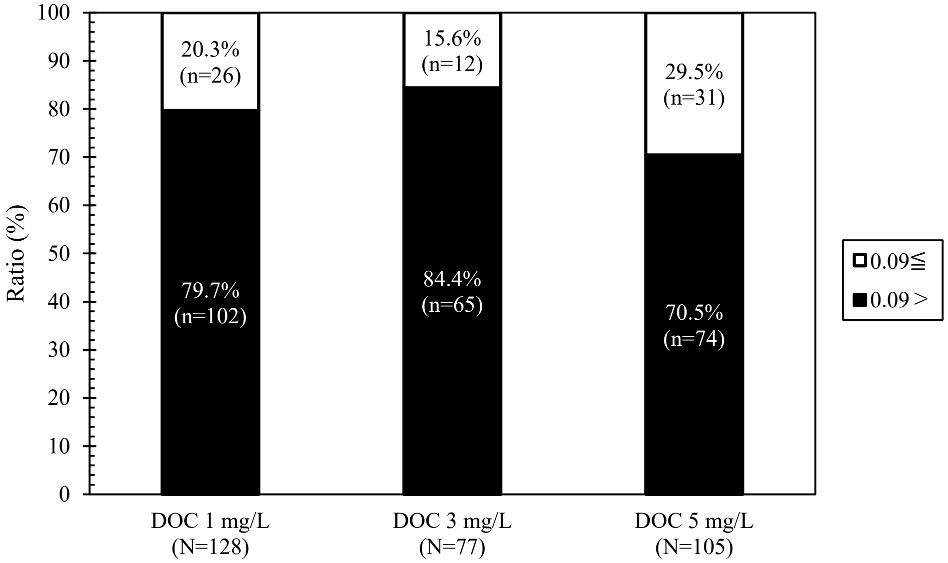

Figure S3. Analytical sample ratio of ≥ 0.09 ng/L and < 0.09 ng/L diss-MeHg with each DOC concentration based on the 310 analytical data points obtained from the incubation experiment.
